# Costs and cost-effectiveness of HIV counselling and testing modalities in Southern Mozambique

**DOI:** 10.1186/s12962-022-00378-9

**Published:** 2022-09-06

**Authors:** Jun Hao Choo, Elisa Lopez-Varela, Laura Fuente-Soro, Orvalho Augusto, Charfudin Sacoor, Ariel Nhacolo, Stanley Wei, Denise Naniche, Ranjeeta Thomas, Elisa Sicuri

**Affiliations:** 1 Department of Infectious Disease Epidemiology, School of Public Health, Faculty of Medicine, Imperial College London, London, UK; 2 ISGlobal, Hospital Clínic—Universitat de Barcelona, Barcelona, Spain; 3 Centro de Investigação Em Saúde de Manhiça (CISM), Maputo, Mozambique; 4 Centers for Disease Control (CDC), Center for Global Health (CGH), Division of Global HIV & TB (DGHT), Maputo, Mozambique

**Keywords:** Acquired immunodeficiency syndrome, Mozambique, Cost–benefit analysis, Decision trees, Uncertainty, Counseling, Health resources

## Abstract

**Objective:**

Despite the high HIV associated burden, Mozambique lacks data on HIV counselling and testing (HCT) costs. To help guide national HIV/AIDS programs, we estimated the cost per test for voluntary counselling and testing (VCT) from the patient’s perspective and the costs per person tested and per HIV-positive individual linked to care to the healthcare provider for VCT, provider-initiated counselling and testing (PICT) and home-based testing (HBT). We also assessed the cost-effectiveness of these strategies for linking patients to care.

**Methods:**

Data from a cohort study conducted in the Manhiça District were used to derive costs and linkage-to-care outcomes of the three HCT strategies. A decision tree was used to model HCT costs according to the likelihood of HCT linking individuals to care and to obtain the incremental cost-effectiveness ratios (ICERs) of PICT and HBT with VCT as the comparator. Sensitivity analyses were performed to assess robustness of base-case findings.

**Findings:**

Based on costs and valuations in 2015, average and median VCT costs to the patient per individual tested were US$1.34 and US$1.08, respectively. Costs per individual tested were greatest for HBT (US$11.07), followed by VCT (US$7.79), and PICT (US$7.14). The costs per HIV-positive individual linked to care followed a similar trend. PICT was not cost-effective in comparison with VCT at a willingness-to-accept threshold of US$4.53, but only marginally given a corresponding base-case ICER of US$4.15, while HBT was dominated, with higher costs and lower impact than VCT. Base-case results for the comparison between PICT and VCT presented great uncertainty, whereas findings for HBT were robust.

**Conclusion:**

PICT and VCT are likely equally cost-effective in Manhiça. We recommend that VCT be offered as the predominant HCT strategy in Mozambique, but expansion of PICT could be considered in limited-resource areas. HBT without facilitated linkage or reduced costs is unlikely to be cost-effective.

## Introduction

Timely HIV diagnosis and linkage to care are essential for improved HIV outcomes [1] but are generally not achieved across sub-Saharan Africa (SSA) [2, 3]. Haber et al. showed that in Kwazulu-Natal, the transition from receiving an HIV-positive diagnosis to care is the weakest in the HIV care continuum [4]. A systematic review reported that, among HIV diagnosed individuals, only one-third of ART-eligible patients were receiving treatment [5]. Cost-effective HIV counselling and testing (HCT) strategies are needed to link more people living with HIV (PLHIV) to care. Few cost-effectiveness analyses (CEAs) on HCT strategies examining linkage to care as an outcome have been conducted, although existing reports have indicated that facility-based strategies are likely more cost-effective than community-based strategies [6, 7].

In Mozambique, where the adult HIV prevalence is among the highest in the world, only 61% PLHIV know their HIV status, and 54% are receiving ART [8]. At the time of this study, Mozambique did not have universal HIV testing [9]. In the country, community-based strategies such as home-based testing (HBT) are infrequently offered [10]. Voluntary counselling and testing (VCT), initiated by the patient at the health facility, has been a mainstay for HCT while provider-initiated counselling and testing (PICT) is the standard approach for healthcare services [9]. In Mozambique as well as in other settings with high burden HIV, PICT lacks optimization strategies in order to reach its full potential [11]. A recent cohort study conducted in Manhiça District found that VCT was associated with the greatest proportion of PLHIV linked to care, followed by PICT and HBT [12]. Importantly, the study procedures did not influence the linkage-to-care beyond the HCT and facility-based referral.

Economic evidence in relation to costs and cost-effectiveness of these HCT strategies in linking PLHIV to care is lacking. By drawing on prospective data from the Manhiça cohort study [12], we estimate the costs of providing HCT strategies in Mozambique and examine the cost-effectiveness of PICT and HBT compared to VCT for initiating linkage to care. In doing so, we compared the level of engagement each HCT strategy entails against the outcome.

In the literature, unit costs of HCT strategies from the provider’s perspective are derived from program costs and usually reported as average cost per person tested and/or average cost per HIV-positive individual identified. The former is more relevant for our study and shall be examined in detail for VCT, PICT, and HBT. Estimates of unit costs per person tested vary. For example, VCT costs in Kenya, Swaziland, Tanzania, and Uganda were reported to be US$8.27–US$28.93 [13–17], and PICT costs were lower (US$5.71–US$11.68) [13–15]. In Kenya, South Africa, and Uganda, several studies reported HBT costs ranging from US$5.00 to US$29.00 per client tested, similar to the range reported for VCT [14, 15, 18–20]. Importantly, costs are not directly comparable across studies because the underlying assumptions and contexts may differ. Findings from our study can help inform policymakers on effective yet affordable national-level HCT that could help identify more PLHIV and link them to care in Mozambique.

## Methods

### Manhiça cohort study

In 2012, HIV prevalence and incidence in Manhiça district were estimated to be 40% and 3.6 infections/100 person-years, respectively [21, 22]. The Manhiça cohort study enrolled 1122 participants with a new HIV-positive diagnosis (May 2014–June 2015), following routine VCT and outpatient PICT at the Manhiça District Hospital and door-to-door HBT by trained healthcare workers (Appendix  1). VCT and PICT resulted in significantly higher proportions of PLHIV linked to care, defined as enrollment in care at the reference district hospital and with a CD4 count registered within the first 3 months after HIV diagnosis, than HBT. Nonetheless, HBT reached a distinct population living in extreme poverty who were in greatest need for facilitated linkage interventions. Details of the study methods and procedures have been described elsewhere [12].

### Cost estimate methods

We employed a micro-costing (bottom-up) approach [23] to estimate the financial and economic costs of HCT provision incurred in the cohort study from the provider’s perspective. As VCT and PICT were routinely offered to the public at the time of the study, estimated costs were referenced from routine health system costs. HBT-associated costs were assessed separately as HBT was not a routine testing strategy. Nevertheless, we examined only recurrent costs and not start-up costs for HBT as there was already an existing structure for carrying out door-to-door interventions e.g. community vaccination.

To determine financial costs, we processed data on capital (e.g. buildings, equipment, and vehicles) and recurrent (personnel, supplies, test-kits, and transport) costs collected May 2014–June 2015. We utilized time and motion data to determine the financial costs of personnel and transport. Time data tracked the time taken by counsellors to travel to and between participants’ houses during HBT and to conduct each counselling session either at the District Hospital or at each participant’s house. On the other hand, motion data included travelling speeds to determine fuel costs. We did not include training costs because HCT in the cohort study was provided by healthcare workers who were not specially trained.

The cost per VCT attendance from the patient’s perspective was determined by a similar micro-costing approach using self-reported explicit costs and implicit costs. Implicit costs were calculated by valuing the patients’ time spent travelling to and waiting at the VCT facility using the average monthly minimum wage in Mozambique, expressed as an hourly wage and assuming an average of 176 work hours (22 days × 8 h/day) per month. We assumed patients would not incur any additional costs for HBT and PICT.

### Data sources

A questionnaire was used to elicit demographic information (Appendix 2). From the patient’s perspective, data were collected from patients accessing VCT by using a specific questionnaire (Appendix 3). From the provider perspective, data were collected from VCT, HBT, and PICT using a separate questionnaire (Appendix 4). Some of the provider resources used (e.g. time to perform the test) were collected for all patients receiving the test (as their HIV status was not known a priory), but only HIV-positive patients were retained for ethical reasons. Where information on costs was not available through questionnaires (e.g. building, furniture, and operation and maintenance costs), we approximated them as a percentage of total recurrent costs based on results from a study by Mwenge et al. [24] in Malawi and Zimbabwe, which share similar sociodemographic and economic profiles as Mozambique [25, 26].

### Data analysis

For cost estimate analysis, we included data on all individuals who tested HIV positive (n = 1277), including non-enrolled individuals (n = 155) who had received a HIV-positive test result previously in a concomitant study. These 155 patients were tested using the same procedure as with the other participants in the main cohort study and were included in our sample size. Of the 1277 patients, 350 were enrolled from VCT and were included in cost estimates from the patients’ perspective. Time data for the following variables were highly negatively skewed due to measurement errors during data collection: travelling to individual houses, performing the test, waiting for test results, and explaining the results. We deemed the 85th percentile to be an appropriate upper limit to capture a reasonable proportion of these data without including outlying values, replacing observations above this percentile with the value at the 85th percentile. All data analyses were conducted using Microsoft Excel 2016 (V.16.14.1) and Stata (V.13.1: Stata Corporation).

We performed descriptive statistical analysis to compute mean (with standard deviation) and median (with maximum and minimum ranges) costs per person tested positive from both the patient’s and provider’s perspective. All costs were expressed in 2015 US$ at an exchange rate of 33.00 Mozambique New Metical (MT) per US$ [27].

### Determination of cost per individual tested

The cost of testing each individual was determined by summing costs of each individual resource, obtained by multiplying the quantities of each resource expended with their approximated unit costs (Appendix 5).

Implicit costs to patients were calculated by valuing their time spent travelling to and waiting at the VCT facility, using the average monthly minimum wage in Mozambique [28]. Capital costs were factored as a proportion of total costs. Fuel costs were calculated using motion data assuming an average travelling speed of 50 km/hour and an average fuel consumption of 0.143 L/km, based on 2013 estimates from the Global Fuel Economy Initiative [29].

### Determination of HCT costs and cost-effectiveness

A decision tree model (Appendix 6) was designed for the CEA to determine cost-effectiveness ratios (CERs) and incremental cost-effectiveness ratios (ICERs). We defined effectiveness as linkage to care based on the cohort study’s definition. Our primary outcome measure was incremental cost per enrolled individual linked to care. The model’s time horizon of 1 year mirrored the duration of the cohort study.

The model captured all steps of the HIV care cascade for both VCT and PICT from diagnosis to retention in care after 12 months of follow-up. For HBT, the model additionally captured steps before the HCT process, from being reached through HBT to obtaining consent. One-year cumulative proportions, reported as the percentage uptake at each step of the HIV care cascade (figures reported in Appendix 1), were directly converted into conditional probabilities for parameterizing the decision tree (Table 1).

**Table 1:** Base-case inputs of parameters (probabilities and costs) for the model, their candidate distributions, and alpha–beta values characterizing those distributions (Manhiça District, Mozambique)

Model inputs	PICT	HBT	VCT	Distribution^a^	Alpha-Beta^b^	Source
Probabilities^c^						
Individual reached	**–**	0.752	**–**	β	8192–2705 (HBT)	[12]
Not known HIV-positive/not pregnant	**–**	0.822	**–**	β	6736–1456 (HBT)	[12]
Gave consent^d^	**–**	0.760	**–**	β	5116–1620 (HBT)	[12]
HIV test positive	0.312	0.072	0.119	β	1046–2305 (PICT); 369–4747 (HBT); 909–6718 (VCT)	[12]
Enrolled in study	0.404	**–**	0.363	β	423–623 (PICT); 330–579 (VCT)	
Enrolled in care	0.908	0.355	0.985	β	384–39 (PICT); 131–238 (HBT); 325–5 (VCT)	[12]
Attended 1st consultation	0.799	0.901	0.911	β	307–77 (PICT); 118–13 (HBT); 296–29 (VCT)	[12]
Linked to care	0.642	0.746	0.693	β	197–110 (PICT); 88–30 (HBT); 205–91 (VCT)	[12]
Costs^e^						
Average cost per test (SD), US$	7.14 (1.30)	11.07 (3.82)	7.79 (1.30)	γ	30.3–0.235 (PICT); 8.39–1.32 (HBT); 36.1–0.216 (VCT)	Table 2

^a^Candidate distribution were determined based on expert opinion and other similer studies

^b^Alpha-Beta values were dependent on the type of candidate distribution. For β distribution, alpha = number of people reaching that step while beta = total number of people who completed previous step – alpha. For γ distribution, alpha = $\frac{{\mu^{2} }}{{s^{2} }}$ while beta = $\frac{{s^{2} }}{\mu \prime }$ where μ is the mean and *s*^2^ is the variance

^**c**^These were conditional probabilities derived directly from proportions of individuals reaching each step over the number of individuals completing the previous step, as described in the cohort study

^d^The probability of giving consent to be tested is a proxy to HBT uptake. Moreover, step downstream are conditional on successfully obtaining consent

^**e**^Average costs per person tested determined for each strategy from the micro-costing analysis were fed into the model. These were assumed to be equivalent to the average cost per test

*PICT* provider-initiated counselling and testing, *HBT* home-based testing, *VCT* voluntary counselling and testing, *SD* standard deviation

The key assumptions of the model were:

1. Linear, unidirectional transitions along the care cascade; uptake at each step must be conditional on the previous step;
2. Only early treatment uptake (≤ 3 months post-diagnosis) at each step was modelled; and.
3. Costs incurred for individuals who cannot be reached through or deemed ineligible for HBT are inconsequential to total program costs.

We calculated the expected average costs and linkage-to-care proportion per person tested for each HCT strategy, by summing costs and outcomes of each branch of the tree weighted by their associated probabilities of occurrence. A ratio of average costs and average expected linkage-to-care proportion was calculated to determine expected HCT linkage-to-care costs.

$${\text{HCT cost per person linked to care}} = \frac{Average \,cost\,per\,test}{{Expected\,proportion\,linked\,to\,care\,per\,test\,among\,all\,who\,were\,tested}}$$

Unlike the derivation of CERs, data only for enrolled individuals was used to determine ICERs to facilitate accurate comparisons of effectiveness. We determined ICERs by dividing the difference in expected costs over the difference in the expected proportion of enrolled individuals linked to care.

$${\text{ICER }} = \frac{{Average\,cost\,per\,test\,\left( {PICT/HBT} \right) - Average \,cost\,per\,test\,\left( {VCT} \right)}}{{Expected\,enrolled\,proportion\,linked\,to\,care\,\left( {PICT/HBT} \right) - Expected\,enrolled\,proportion\,linked\,to\,care\,\left( {VCT} \right)}}$$

### Sensitivity analysis

Our univariate sensitivity analysis varied key cost-related parameters within predefined sensitivity ranges (Appendix 7) to explore the robustness of results. Lacking published data, we empirically adjusted base-case values by ± 20%, referencing ranges used by Mwenge et al. in their determination of HCT costs in three SSA countries [24].

We performed deterministic and probabilistic sensitivity analyses (DSA and PSA) as part of the CEA. In the DSA, probabilities and average costs per test were varied at the maximum and minimum values defined by their respective distributions (Table 1). In the PSA, we performed 2000s-order Monte-Carlo simulations and plotted each result on a cost-effectiveness plane. Cost-effectiveness acceptability curves (CEACs) were constructed to determine the cost-effectiveness threshold.

## Results

### Costs

#### Costs to the provider

Base-case median costs were higher for VCT per individual tested (US$7.82; range, US$4.54–US$15.14) than for PICT per individual tested (US$7.29; range, US$2.30–US$14.67) but were lower than HBT per individual tested (US$11.62; range, US$4.32–US$19.83; Table 2). The small difference in costs between the two facility-based strategies (VCT & PICT) can be attributed to higher personnel costs for VCT which probably resulted from a longer time spent on HCT. As expected, HBT costed more than facility-based strategies, driven by higher personnel costs and transport costs. Like VCT and PICT costs, cost component proportions of HBT costs were almost equal: personnel (32.2%), supplies (36.6%) and transport (29.6%). Resources used are reported by HCT strategy in Appendix 8.

**Table 2:** Breakdown of costs to the provider per individual tested for all 3 HCT strategies among individuals in the cohort study who tested HIV-positive

Cost item	VCT (*n* = 350)		PICT (*n* = 455)		HBT (*n* = 472)	
	Average cost/person tested (SD), US$	Median cost/person tested (range), US$	Average cost/person tested (SD), US$	Median cost/person tested (range), US$	Average cost/person tested (SD), US$	Median cost/person tested (range), US$
Capital costs^a^						
Building, furniture	0.25 (0.04)	0.26 (0.15–0.49)	0.23 (0.04)	0.24 (0.07–0.48)	–	–
Recurrent costs						
Personnel	3.11 (1.22)	3.12 (0.56–10.08)	2.49 (1.20)	2.62 (00.50–9.64)	3.64 (1.00)	3.80 (1.03–7.32)
Supplies	4.32 (0.00)	4.32 (4.32–4.32)	4.32 (0.35)	4.32 (4.27–4.36)	4.39 (0.57)	4.32 (3.21–9.68)
O & M^b^	0.12 (0.02)	0.12 (0.07–0.24)	0.11 (0.02)	0.12 (0.04–0.23)	–	–
Transport (fuel)	–	–	–	–	3.04 (2.82)	3.50 (0.00–7.33)
Total recurrent costs	7.55 (1.23)	7.56 (4.32–14.40)	6.91 (1.23)	7.06 (2.18–13.96)	11.07 (3.82)	11.62 (4.32–19.83)
Total costs (capital and recurrent)	7.79 (1.30)	7.82 (4.54–15.14)	7.14 (1.30)	7.29 (2.30–14.67)	11.07 (3.82)	11.62 (4.32–19.83)

All figures are reported to 2 decimal places. SD refers to standand deviation

^a^This was approximated as a percentage (3.27%) of the totel costs. Building and furniture costs were not annuitised

^b^This was approximated as a percentage (1.65%) of the totel recurrent costs

*PICT* provider-initiated counselling and testing, *HBT* home-based testing, *VCT* voluntary counselling and testing, *SD* standard deviation, *O & M* operation and management

#### Costs to the patient

The median base-case total cost to the patient per VCT visit was US$1.08 (range, US$0.00–US$19.58). A breakdown of total costs into explicit (median, US$0; range US$0–US$18.62) and implicit costs (median, US$0.86; range, US$0.00–US$4.65) showed most costs (~ 80%) were implicit, which reflected waiting times and travelling times to the hospital (Appendix 9).

#### Sensitivity analysis

Average and median costs of VCT and PICT from the provider’s perspective were most sensitive to variations in operation and maintenance, and capital costs (Appendix 10). However, closer examination revealed this was largely a result of high estimates obtained from reports in Kenya and Uganda, which were unlikely to represent real costs in Mozambique [13, 14].

Although unit costs of VCT and PICT were less sensitive to test-kit prices, the large extent of overlap between them in the sensitivity analysis suggested that accurate determination of these prices is crucial, since the order of costs could differ should assumptions change. On the other hand, HBT costs were most sensitive to the choice of percentile cut-off for the upper limit of several time-related variables. Median HBT cost was especially sensitive to the low estimate, varying from the base-case value by 41%. Base-case average and median VCT costs from the patient’s perspective were generally robust (Appendix 11).

### HCT costs and cost-effectiveness of HCT to link to care

#### Base-case results

The costs per HIV-positive individual linked to care, covering both testing and linkage cost, were estimated at US$289.67 for VCT, US$121.46 for PICT, and US$643.37 for HBT (Table 3). PICT was less expensive than VCT but also resulted in a lower proportion of PLHIV linked to care. As PICT is less costly but also less efficacious than VCT, PICT ICER of US$4.15 could be interpreted as savings per indivual not linked to care. PICT falls in the low cost/worse outcome quadrant of the cost-effectiveness plane (Appendix 12). In comparison with VCT, the HBT ICER (−US$8.57) was in the high cost/worse outcome quadrant.

**Table 3:** Base-case results of HCT costs and cost-effectiveness comparisons between PICT & VCT and HBT & VCT

	VCT	PICT	HBT
HCT costs			
Average cost per test^a^, US$	7.79	7.14	11.07
Expected proportion of HIV^+^ individuals linked to care among those tested	0.0269	0.0588	0.0172
Cost per HIV^+^ individual linked to care, US$	289.67	121.46	643.37
Cost-effectiveness comparisons			
Effect–Expected proportion of HIV^+^ individuals linked to care among those enrolled only	0.621	0.466	0.238
Incremental cost, US$	–	−0.65	3.28
Incremental effect	–	−0.155	−0.383
ICER	–	4.15	−8.57 (Dominated)^b^

All costs are reported to 2 decimal places. All other figures are reported to 3 significant figures

^a^Assume cost per person tested positive is equivalent to cost per test

^b^A dominated scenario occurs when the comparator, in this case VCT, is less costly but more effective than HBT. HBT is said to be dominated by VCT

*PICT* provider-initiated counselling and testing, *HBT* home-based testing, *VCT* voluntary counselling and testing, *ICER* incremental cost-effectiveness ratio

#### Sensitivity analyses

HCT costs per individual linked to care were most sensitive to the average cost per test (Appendices 13, 14). However, within the narrower ranges elicited from the sensitivity analysis for unit costs, base-case linkage-to-care costs for VCT and PICT were robust. Unfortunately, there was still great uncertainty in the determined HCT costs of HBT.

**Fig. 1:**
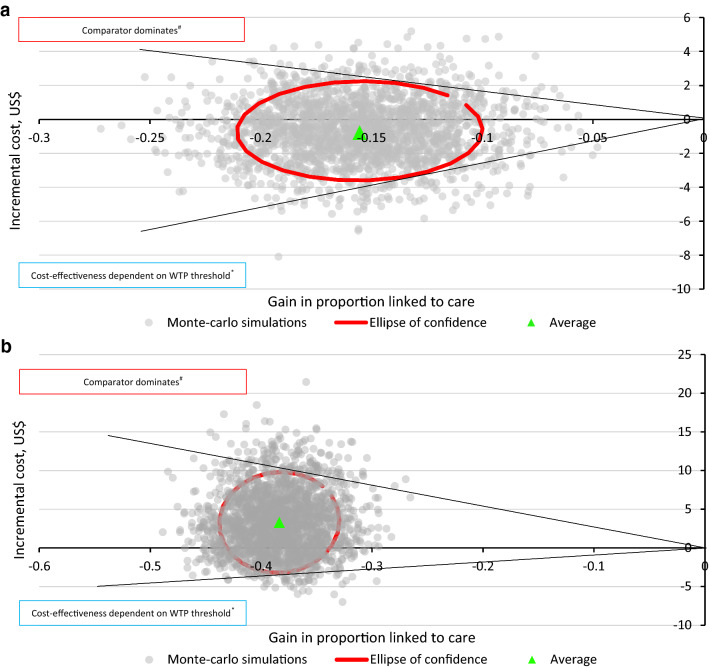
Plots of 2000s-order Monte-Carlo simulations of incremental cost per proportion linked to care gained on high cost/worse outcome and low cost/worse outcome quadrants of the cost-effectiveness plane. **a** Monte-Carlo simulations for the comparison between PICT and VCT; **b** Monte-Carlo simulations for the comparison between HBT and VCT. *Interpretation of ICERs in the low cost/worse outcome quadrant is dependent on the WTA threshold. ^#^ICERs in the high cost/worse outcome quadrant represent a dominated scenario for the strategy under comparison. *Note:* The red ellipse of confidence encircles 95% of the bootstrapped ICERs, and black solid lines represent quasi 95% confidence intervals **a** ICERs have almost equal chance of falling into the high cost/worse outcome and low cost/worse outcome quadrants. **b** ICERs are more likely to fall in the high cost/worse outcome quadrant. PICT provider-initiated counselling and testing, HBT home-based testing; VCT voluntary counselling and testing, ICER incremental cost-effectiveness ratio, WTA willingness to accept

Similarly, ICERs were most sensitive to unit costs per test although they too were fairly robust within the narrower ranges obtained from the sensitivity analysis for unit costs (Appendix 15). Both the DSA and PSA showed significant uncertainty in determining the cost-effectiveness of PICT in relation to VCT, notwithstanding considerations of the cost-effectiveness threshold. HBT, however, was likely a dominated strategy. The probabilistic results were similar to the deterministic findings (Fig. 1).

**Fig. 2:**
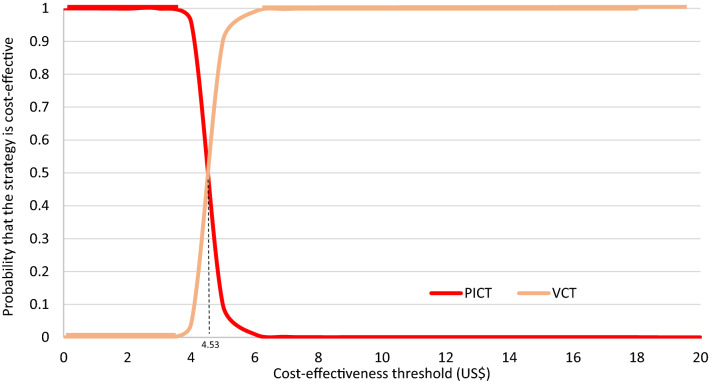
CEAC of the comparison between PICT and VCT. The interpretation of ICERs in the low cost/worse outcome quadrant is dependent on the cost-effectiveness threshold or the minimum WTA. PICT was initially the most cost-effective strategy up until a WTA of US$4.53. At higher WTA, VCT was undoubtly the more cost-effective strategy. *CEAC* cost-effectivenss acceptability curves, *PICT* provider-initiated counselling and testing, *VCT* voluntary counselling and testing, *ICER* incremental cost-effectiveness ratio, *WTA* willingness to accept

The interpretation of ICERs in the low cost/worse outcome quadrant for the PICT comparison is dependent on the minimum willingness-to-accept (WTA) for every individual successfully linked to care. As there was no clearly defined WTA threshold, a CEAC was constructed (Fig. 2). Since the elicited threshold of US$4.53 was greater than the base-case and average ICER in the PSA, we concluded PICT was not cost-effective relative to VCT, although this result was only marginal. Moreover, both the DSA and PSA indicated significant uncertainty in this result. Interestingly, both strategies had almost equal likelihood of being cost-effective when the cost-effectiveness threshold was mapped on the Monte-Carlo plot (Appendix 16). A CEAC was not produced for the comparison between HBT and VCT because HBT was clearly dominated (high cost/worse outcome) at all cost-effectiveness thresholds above zero, i.e., the corresponding CEAC is a flat horizontal line at y = 0.

## Discussion

To our knowledge, this is the first study in Mozambique to estimate costs and cost-effectiveness of VCT, PICT, and HBT. We found the median cost per VCT attendance to be as low as US$1.00, largely comprising implicit costs. This is commensurate with VCT prices and patients’ willingness-to-pay reported elsewhere [34, 35], suggesting that the costs to patients were reasonable. However, differences in health systems, wage structure, and socioeconomic conditions between countries may lead to different valuations of explicit and implicit costs.

We found the average cost to the provider per individual tested and derived costs per HIV-positive patient linked to care to be greatest for HBT, followed by VCT and PICT. Both the magnitude and trend of costs elicited were mostly consistent with the available literature for neighboring SSA countries (in 2009 prices), with the exception of the study by Menzies et al. (in 2007 prices) [13–15]. Notwithstanding considerations for effectiveness, PICT is the cheapest for potential HCT scale-up in areas with inadequate coverage. However, in resource-limited Mozambique, HCT scale-up requires more than mere consideration of HCT costs as improvement of existing healthcare infrastructure and expansion of the healthcare workforce are also needed.

We found HBT to be substantially more expensive than VCT and PICT. This was not consistent with the available literature. Five studies across Uganda, Kenya, and South Africa reported HBT costs ranging from US$5.00–US$29.00 per client tested that were in several cases lower than reported VCT and PICT costs [14, 15, 18–20]. A difference in cost analysis approach likely accounts for this discrepancy, since reported cost estimates from a program perspective are more likely to capture economies of scale accurately, particularly for HBT. Another reason could be the numerous far-lying residential areas in Manhiça [21], which probably required substantial resources for HBT.

CEA results suggested PICT was not cost-effective to link PLHIV to care, relative to VCT; more precisely, PICT was less expensive but also less effective than VCT. However, this result was only marginal. Moreover, the lack of robustness in the base-case results of the CEA comparing PICT and VCT, coupled with the fact that PICT was consistently reported to be cheaper than VCT [13, 15] yet with almost comparable linkage-to-care proportions [6, 36], suggests a high possibility that PICT could be considered cost-effective as well, especially in areas with limited resources. PICT could be considered for expansion and scale-up, depending on the context and available resources, in line with regional trends [37–40]. For example, in remote areas where health facilities may be understaffed, PICT could be expanded to include the general population to lower costs of HCT but still yield reasonable linkage-to-care proportions. Conversely, in areas with more resources and likely higher WTA, PICT could be limited.

As expected, HBT was more expensive and had a lower expected linkage-to-care proportion than VCT. This was consistent with several studies [19, 41–46]. However, other reports showed high linkage-to-care proportions for HBT (47.5%–70.0%) [47–51]. Although definitions of linkage to care were inconsistent in the literature, Kiene et al. highlighted an obvious but important trend: HBT programs that facilitate linkage perform better than those that do not [52]. Sharma et al.’s systematic review of 126 studies confirmed this trend, showing high linkages to care for strategies that facilitated linkages [6]. Similarly, Gilbert et al. found community-based TB and HIV integrated screening and linkcage to care strategies to be cost-effective in South Africa [53]. Moreover, the Manhiça cohort study investigators also hypothesized that strategies to facilitate linkage to care were important for cost-effectiveness [12]. HBT could be cost-effective if integrated with facilitated linkage strategies.

Our study had limitations. Because data were only available for PLHIV, we modelled total program costs. The inability to determine real program costs may limit the usefulness of our findings for policymakers [54]. Moreover, the lack of methodological consistency between our study and published studies may result in overestimates and bias cost comparisons. In Mozambique, some HIV-testing costs are borne by the government and others by international donors; our study did not distinguish between the two. In addition, analysis of costs to the provider did not include other costs, such as training, supply chain, and program management. HBT, as analyzed in this study, referred to door-to-door universal testing, and results cannot be extrapolated to other forms of community testing, such as index case testing, which is prominent in SSA testing programs.

Results from this study are hardly generalizable to other countries. Manhiça is a small, semi-rural district in Mozambique where HCT services are offered free-of-charge at district hospitals. It is unclear how the imposition of charges, like in Kenya and Tanzania [34, 35], would influence HCT uptake and linkage-to-care. The representativeness and precision of this study’s findings may also be limited since only data from a single cohort study were used, and findings are subject to limitations in that study. For example, López-Varela et al. acknowledged that attrition in linkage to care in the cohort study could have been overestimated due to poor record keeping. However, such overestimation is likely lower than in other studies because the authors used data from the Health and Disease Surveillance System [12].

Because our dataset lacked detailed cost information, we were unable to evaluate the combination of VCT with either PICT or HBT against standalone VCT. Future studies, however, could investigate such combinations and provide information to policymakers on how best to improve existing HIV-testing policies in Mozambique.

A CEA may be unsuitable for assessing the utility of HBT to meet targets for HIV eradication. CEAs are often designed to maximize efficiency at the expense of achieving distributive equality and equity [55]. Therefore, active HCT approaches, such as HBT, are seldom favored over more passive strategies due to their high unit costs per outcome achieved. Nevertheless, such strategies play a crucial role in achieving HIV eradication. HBT might not be cost-effective but may be equitable because HBT can reach populations distinct from those reached by facility-based strategies [12]. CEAs can address health equity concerns either through an equity impact analysis or an equity trade-off analysis as introduced by Cookson et al. [56] Round et al.’s analytical framework, which uses equity weights in decision analyses, may be useful [57]. Addressing equity is relevant for HCT strategies because factors like age, sex, and socioeconomic status may result in differential rates of uptake and linkage to care [12, 58, 59].

Despite these limitations, this study generates, for the first time, information for HIV healthcare policy decision making in Southern Mozambique, a setting characterized by one of the highest community based HIV prevalences in the world (i.e., 40%). From the research perspective, this project calls for the need to test the cost-effectiveness of comprehensive home-based strategies including not only screening but also linkage to care and promotion of ART adherence. A similar combination prevention approach involving universal HIV testing and treatment has recently been assessed to be a cost-effective strategy at thresholds greater than US$800 per DALY averted in Zambia and South Africa [60]. This may be seen as a reasonable threshold also for Mozambique, considering the traditional threshold of three times the gross domestic product (GDP) per capita and that the GDP per capita of Mozambique is just below US$500. However, site specific studies should be carried out to obtain valuable information.

## Figures and Tables

**Fig. 3:**
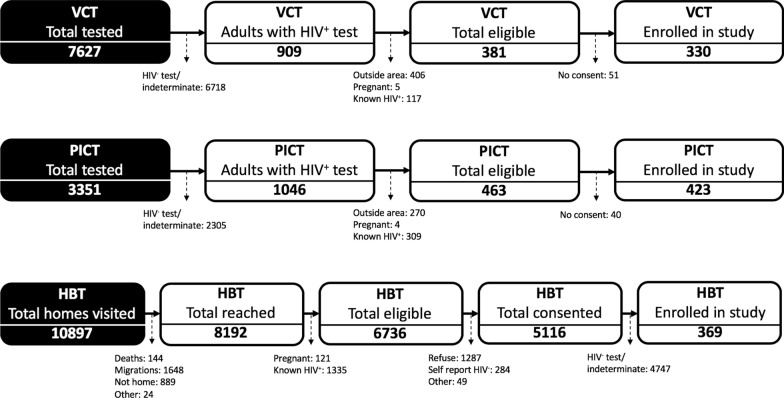
Steps involved in the enrollment of participants into the study across all three HIV counselling and testing strategies. [16] Attrition at each step was quantified. *VCT* voluntary counselling and testing, *PICT* provider-initiated counselling and testing, *HBT* home-based testing

**Fig. 4:**
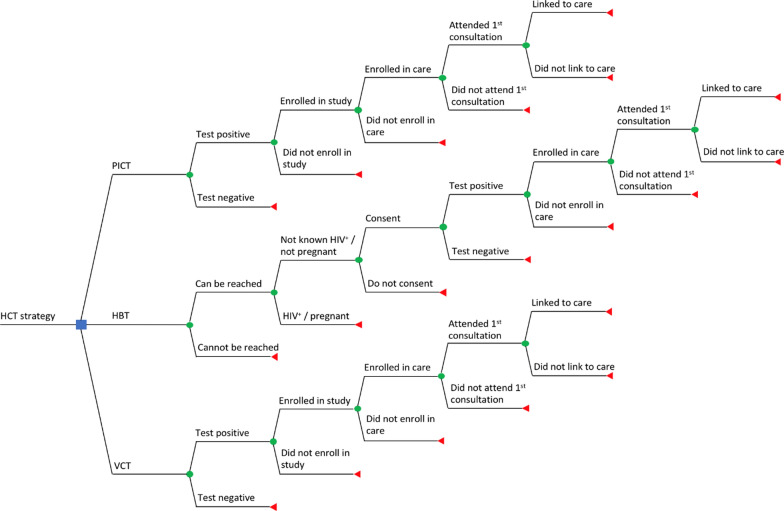
Each branch depicts individual steps of the HIV care cascade as described in the cohort study and is conditional on the previous step. The blue square node represents a decision node, green circular nodes represent chance nodes, and red triangles illustrate terminal nodes. *HCT* HIV counselling and testing, *PICT* provider-initiated counselling and testing, *HBT* home-based testing, *VCT* voluntary counselling and testing

**Fig. 5:**
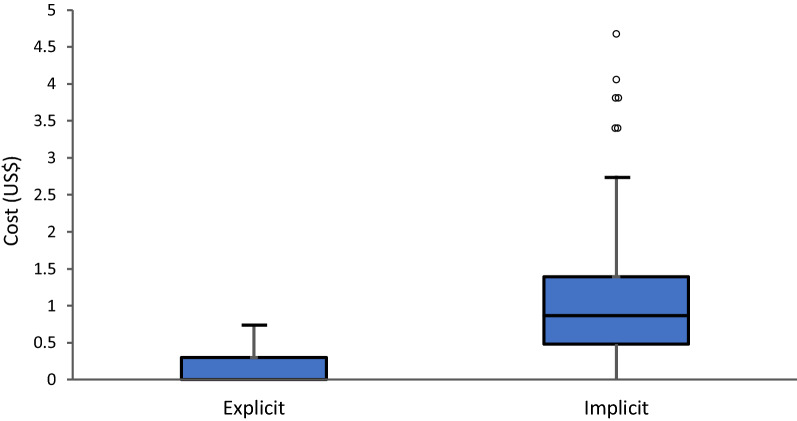
Total cost was driven largely by implicit costs. It can thus be inferred that transport, food and drink costs were insignificant in comparison to opportunity costs of waiting and travelling times. Two anomalous values for explicit cost are not shown: 12.76 and 18.62. Values for implicit costs outside the interquartile range: 3.46, 3.74, 4.03 and 4.65. The value of zero for implicit costs reflect minimal travelling and waiting times, which was the case for a couple of patients

**Fig. 6:**
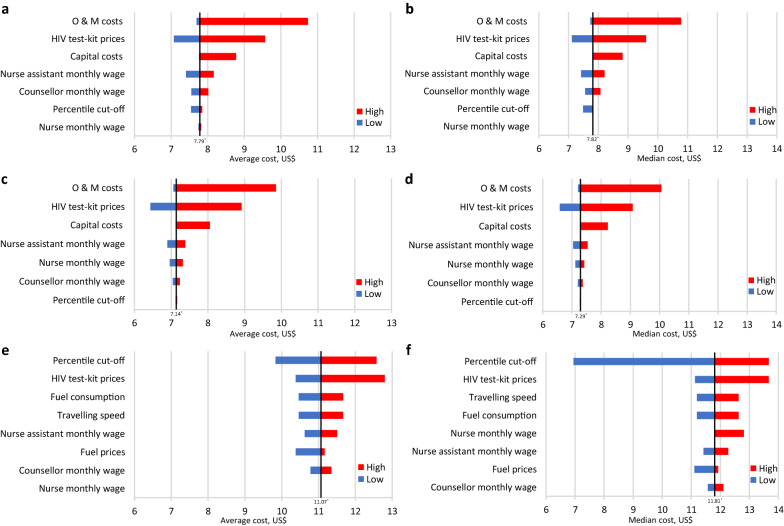
Tornado plots of average and median costs per person tested when assumptions were varied between the high and low estimates. **a** Average costs of VCT, **b** Median costs of VCT, **c** Average costs of PICT, **d** Median costs of PICT, **e** Average costs of HBT, **f** Median costs of HBT. Varying nurses’ monthly wage did not affect median cost of VCT and average cost of HBT. *Black vertical lines represent base-case costs

**Fig. 7:**
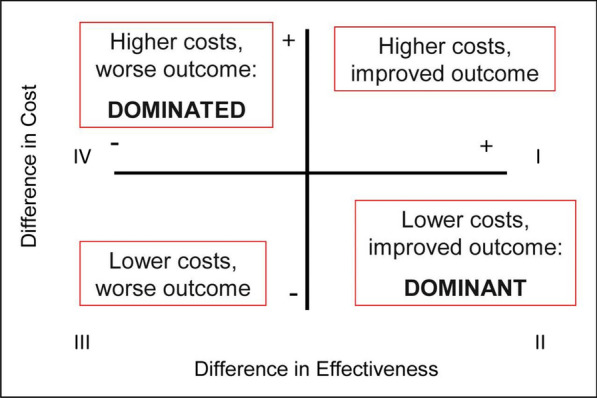
ICERs in quadrants I and III represent scenarios in which the cost-effectiveness of the strategy under comparison is dependent on the cost-effectiveness threshold. Abbreviations: ICER, incremental cost-effectiveness ratios

**Fig. 8:**
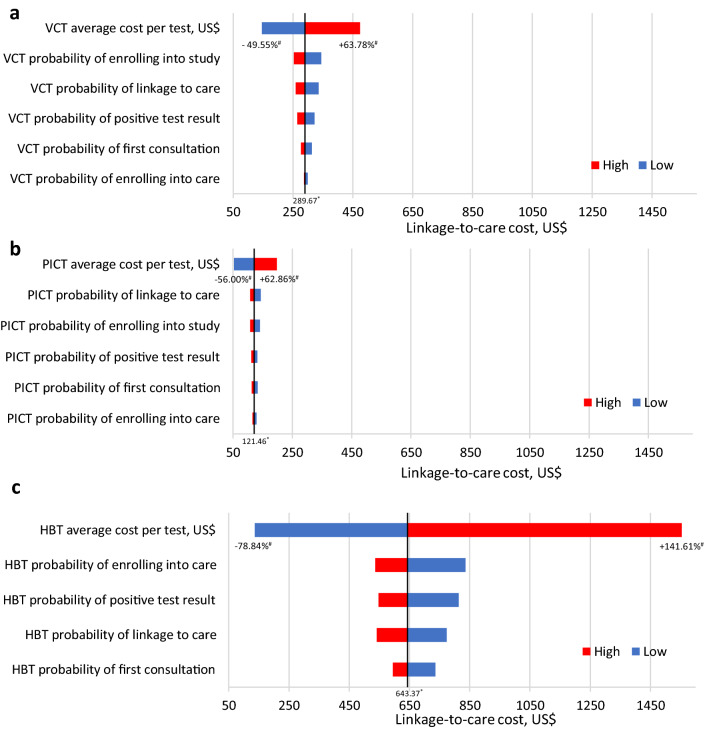
**a** Linkage-to-care costs of VCT, **b** Linkage-to-care costs of PICT, **c** Linkage-to-care costs of HBT. *Black vertical lines represent base-case results. ^#^Percentage change from base-case costs quantifies the impact of varying cost per test between strategies. *VCT* voluntary counselling and testing, *PICT* provider-initiated counselling and testing, *HBT* home-based testing

**Fig. 9:**
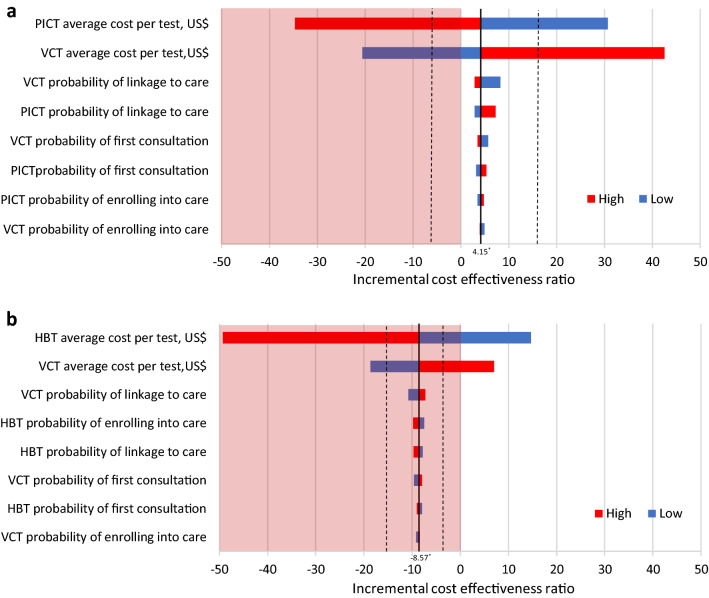
**a** ICERs for the comparison between PICT and VCT, **b** ICERs for the comparison between HBT and VCT. *Black vertical solid lines represent base-case results. *Note:* the area shaded red covers all negative ICERs and represents a dominated scenario by VCT for the strategy under comparison. Black dotted lines demarcate the range of ICERs for each comparison that would result from the corresponding ranges of HCT unit costs. **a** The shaded area between the lower black dotted line and the origin represents the likelihood that base-case ICER was not robust and a dominated scenario for PICT. **b** The area enclosed by both dotted lines lie within the shaded region, suggesting base-case findings that HBT was dominated were robust up to this range of unit costs. *VCT* voluntary counselling and testing, *PICT* provider-initiated counselling and testing, *HBT* home-based testing, *ICER* incremental cost-effectiveness ratios

**Fig. 10:**
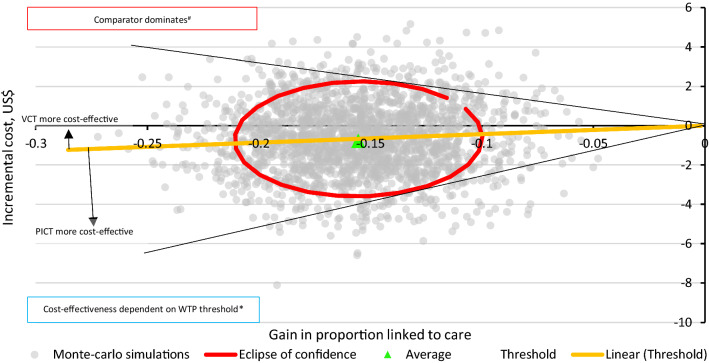
*ICERs in the south-west quadrant depict when the cost-effectiveness of PICT is dependent on the WTA threshold. ^#^ICERs in the north-west quadrant represent a dominated scenario for PICT. The red ellipse of confidence encircles 95% of the bootstrapped ICERs while black solid lines represent quasi 95% confidence intervals. There is an almost equal chance for ICERs to fall either side of the threshold within the region bounded by the quasi 95% confidence intervals, implying that PICT have equal likelihood of being cost-effective or not relative to VCT*. WTA* willingness-to-accept, *VCT* voluntary counselling and testing, *PICT* provider-initiated counselling and testing, *ICER* incremental cost-effectiveness ratio

## Data Availability

The datasets used and/or analysed during the current study are available from the corresponding author on reasonable request.

## Appendices

### Appendix 1: Schematic of cohort study with figures used in the economic evaluation

Numbers in Fig. 3 are used in the cohort decision model presented in Fig. 1.

### Appendix 2: Copy of the questionnaire administered to study participants to elicit demographic information

Questionnaire was administered in Portuguese.

### Appendix 3: Copy of the questionnaire administered to study participants to elicit cost-related information for voluntary counselling and testing (VCT) from the patient’s perspective

Questionnaire was administered in Portuguese.

### Appendix 5: Unit costs (US$) of various resource categories employed in the micro-cost estimate approach, along with their low and high estimates as determined from individual sources (Manhiça District, Mozambique)

**Table:**

Resource	Unit cost, US$	Low estimate, US$	High estimate, US$	Source
Patient’s perspective				
National monthly minimum wage (for agriculture and fisheries)^a^	101.35	99.04	114.56	[28]
Provider’s perspective				
Determine HIV rapid test kit	1.07	0.98	1.16	[30]
Unigold HIV rapid test kit	3.18	2.59	4.79	[30]
Lancets	0.02	No data	No data	CISM private communication^b^
Gloves	0.01	No data	No data	[31]
Pipettes	0.04	No data	No data	[32]
Nurse monthly wage	792.00	No data	No data	CISM private communication^b^
Nurse assistant monthly wage	400.00	No data	No data	CISM private communication^b^
Counsellor monthly wage	258.32	No data	No data	CISM private communication^b^
Fuel, per litre^c^	1.55	1.20	1.60	[33]

All costs are reported to 2 decimal places.

^a^The monthly minimum wage for agriculture and fisheries best reflect the type of predominant economic activities in Manhiça.

^b^Cost of lancets and montly wages of nurses, nurse assistants and cousellors working at MDH were communicated privately by Centro de Investigaçâo em de Saúde de Manhça (CISM). No other sources of data were available to derive low and high estimates.

^c^Fuel costs bases on fuel prices in 2014.

### Appendix 6: A cohort decision-analytic model (decision tree) comparing HCT strategies for linking HIV-positive patients to care in Manhiça District, Mozambique

See Fig. 4

### Appendix 7: Range of input values explored in the univariate sensitivity analysis for each assumption (input) made in the determination of costs per individual tested

**Table:**

Inputs	Input values			Source of estimates
	Base-case	Low estimate	High estimate	
Patient’s perspective				
Minimum monthly wage, US$	101.35	99.04	114.56	From Appendix 5
Monthly number of working hours	176	141 (−20%)	211 (+ 20%)	Empiric determination^b^
Provider’s perspective				
Percentile cut-off for upper limit of variables	85th	80th	90th	Empiric determination^b^
HIV test kit prices, US$	(Determine = 1.07; Unigold = 3.18)	(Determine = 0.98; Unigold = 2.59)	(Determine = 1.16; Unigold = 4.79)	From Appendix 5
Percentage of total costs as capital costs	3.27	3.00	14.23^a^	[13, 14, 17, 24]
Percentage of recurrent costs as O & M costs	1.65	0.55	28.72^a^	[13, 17, 20, 24]
Nurse monthly wage, US$	792.00	633.60 (−20%)	950.40 (+ 20%)	Empiric determination^b^
Nurse assistant monthly wage, US$	400.00	320.00 (−20%)	480.00 (+ 20%)	Empiric determination^b^
Counsellor monthly wage, US$	258.32	206.66 (−20%)	309.98 (+ 20%)	Empiric determination^b^
2014 fuel prices, US$/l	1.55	1.20	1.60	From Appendix 5
Travelling speed, km/h	50	40 (−20%)	60 (+ 20%)	Empiric determination^b^
Fuel consumption, l/km	0.143	0.114 (−20%)	0.171 (+ 20%)	Empiric determination^b^

^a^These were ambitiously high estimates which unlikely represent true high estimates for Mozambique

^b^When there were no published data for ranges, base-case values were adjusted empirically (± 20%). Base-case values for monthly number of working hours were based on a 22-day work month and average daily working hours of 8 h/day. Base-case values for travelling speed were based on average speed limits in Manhiça

*O & M* operation and management

### Appendix 8: Quantities of resources used in each testing modality

**Table:**

Testing modality	Quantity by test strategy (cost in the case of transportation, food and drinks, and others) Mean values and standard deviation are reported		
	VCT	PICT	HBT
Patient’s perspective			
Transport (bus, taxi, etc.), US$	0.19 (0.35)	–	−
Drink + Food, US$	0 (0.25)	−	−
Waiting time (hours)	0.95 (0.94)	−	−
Travelling time (hours)	0.82 (0.71)	−	−
Any other direct cost, US$	0.1 (1.05)	−	−
“Determine” HIV rapid test kit (number)	1 (0)	1 (0)	1.03 (0.27)
Provider’s perpective			
“Unigold” HIV rapid test kit (number)	1 (0)	1 (0)	1.01 (0.12)
Lancets (number)	1 (0)	1 (0)	1.04 (0.21)
Gloves (number)	1 (0)	1(0)	1.16 (0.38)
Pipettes (number)	1 (0)	1 (0)	1 (0)
Personnel time (minutes)^a^	46.53 (12.64)	30.72 (12.60)	58.45 (15.30)
Kilometers to reach the patient	−	−	13.7 (12.75)

^a^Personnel time was measured through a time and motion study. Personnel included councellors and/or nurses and clinicians in provider-initiated counselling and testing (PICT). Personnel time includes time to reach patient (home-based testing [HBT] only), explanation, doing the test, waiting for results, and explaining the results and next steps

^*VCT* voluntary counselling and testing^

### Appendix 9: Box and whiskers plot of median explicit and implicit costs of voluntary counselling and testing, in US$, to the patient

See Fig. 5.

### Appendix 10: Sensitivity analysis of average and median costs per person tested

See Fig. 6.

### Appendix 11: Impact of varying assumptions on base-case average and median costs of VCT from the patient’s perspective among participants who tested HIV-positive

**Table:**

Parameter	Average cost (SD), US$	Median cost (range), US$
Base-case analysis	1.34 (1.46)	1.08 (0.00–19.58)
Monthly minimum wage^a^		
Low estimate = US$99.04	1.32 (1.45)	1.07 (0.00–19.56)
High estimate = US$114.56	1.47 (1.50)	1.19 (0.00–19.70)
Number of working hours per month^a^		
Low estimate = 141 h	1.59 (1.55)	1.32 (0.00–19.82)
High estimate = 211 h	1.17 (1.40)	0.96 (0.00–19.42)

All costs are reported to 2 decimal places. SD refers to standard deviation

^a^The monthly minimum wage and number of working hours per month in the base-case analysis was US$101.35 and 176 h respectively

*VCT* voluntary counselling and testing, *SD* standard deviation

### Appendix 12: Cost-effectiveness plane depicting the four quadrants in which ICERs may reside

See Fig. 7.

### Appendix 13: Tornado plots illustrating fluctuations in linkage-to-care costs between the minimum (low) and maximum (high) values of individual model parameters in the univariate sensitivity analysis

See Fig. 8.

### Appendix 14: Impact of varying probabilities and costs used to parameterise the decision tree on linkage-to-care costs of VCT, PICT, and HBT from the provider’s perspective, US$

**Table:**

Model parameters	VCT		PICT		HBT	
	Min parameter value^a^	Max parameter value^a^	Min parameter value^a^	Max parameter value^a^	Min parameter value^a^	Max parameter value^a^
Probabilities						
Positive test result	322.24 (+ 11.24%)	263.38 (−9.08%)	132.47 (+ 9.06%)	111.79 (−7.96%)	813.86 (+ 14.40%)	547.15 (−7.53%)
Enrolled into study	344.07 (+ 18.78%)	251.99 (−13.01%)	141.39 (+ 16.41%)	107.69 (−11.34%)	–	–
Enrolled into care	298.50 (+ 3.05%)	285.88 (−1.31%)	130.18 (+ 7.18%)	116.06 (−4.45%)	835.64 (+ 20.24%)	535.66 (−15.97%)
Attended first consultation	312.83 (+ 8.00%)	276.09 (−4.69%)	133.15 (+ 9.62%)	112.75 (−7.17%)	736.03 (+ 26.50%)	594.93 (−14.96%)
Linked to care	335.18 (+ 15.71%)	258.26 (−10.84%)	143.59 (+ 18.22%)	107.47 (−11.52%)	773.58 (+ 29.88%)	540.62 (−16.74%)
Costs						
VCT	146.14 (−49.55%)	474.41 (+ 63.78%)	–	–	–	–
PICT	–	–	53.45 (−56.00%)	197.81 (+ 62.86%)	–	–
HBT	–	–	–	–	136.11 (−78.84%)	1554.44 (+ 141.61%)

All figures are reported to 2 decimal places. Percentages in parentheses denote the percentage change from base-case linkage-to-costs for strategy

^a^Minimum and maximum parameter values as tabulated in Table 2

*VCT* voluntary counselling and testing, *PICT* provider-initiated counselling and testing, *HBT* home-based testing

### Appendix 15: Tornado plots illustrating fluctuations in ICERs between the minimum (low) and maximum (high) values of individual model parameters in univariate sensitivity analysis

See Fig. 9.

### Appendix 16: Mapping the WTA threshold on the Monte-Carlo plot for the comparison between PICT and VCT

See Fig. 10.
